# An X-Band CMOS Digital Phased Array Radar from Hardware to Software

**DOI:** 10.3390/s21217382

**Published:** 2021-11-06

**Authors:** Yue-Ming Wu, Hao-Chung Chou, Cheng-Yung Ke, Chien-Cheng Wang, Chien-Te Li, Li-Han Chang, Borching Su, Ta-Shun Chu, Yu-Jiu Wang

**Affiliations:** 1 Department of Electrical Engineering, National Tsing Hua University, Hsinchu 30013, Taiwan; yueming@gapp.nthu.edu.tw (Y.-M.W.); s101061557@m101.nthu.edu.tw (H.-C.C.); tschu@ee.nthu.edu.tw (T.-S.C.); 2 Tron Future Tech Inc., Hsinchu 300042, Taiwan; cyk@tronfuturetech.com (C.-Y.K.); ccw@tronfuturetech.com (C.-C.W.); ctl@tronfuturetech.com (C.-T.L.); lhc@tronfuturetech.com (L.-H.C.); 3 Department of Electrical Engineering, National Taiwan University, Taipei 10617, Taiwan; borching@ntu.edu.tw

**Keywords:** antenna-in-package (AiP), complementary metal-oxide-semiconductor (CMOS), digital beamforming (DBF), digital array radar, phased array, pulsed radar, radar signal detection, system-on-chip (SoC), transceiver, X-band

## Abstract

Phased array technology features rapid and directional scanning and has become a promising approach for remote sensing and wireless communication. In addition, element-level digitization has increased the feasibility of complicated signal processing and simultaneous multi-beamforming processes. However, the high cost and bulky characteristics of beam-steering systems have prevented their extensive application. In this paper, an X-band element-level digital phased array radar utilizing fully integrated complementary metal-oxide-semiconductor (CMOS) transceivers is proposed for achieving a low-cost and compact-size digital beamforming system. An 8–10 GHz transceiver system-on-chip (SoC) fabricated in 65 nm CMOS technology offers baseband filtering, frequency translation, and global clock synchronization through the proposed periodic pulse injection technique. A 16-element subarray module with an SoC integration, antenna-in-package, and tile array configuration achieves digital beamforming, back-end computing, and dc–dc conversion with a size of 317 × 149 × 74.6 mm^3^. A radar demonstrator with scalable subarray modules simultaneously realizes range sensing and azimuth recognition for pulsed radar configurations. Captured by the suggested software-defined pulsed radar, a complete range–azimuth figure with a 1 km maximum observation range can be displayed within 150 ms under the current implementation.

## 1. Introduction

Phased array technology has evolved over the past few decades. Categorized into analog, subarray digital, and element-level digital topologies [1], phased array systems accomplish spatial filtering and power combination through the synchronous excitation of each radiating element. In particular, element-level digitization provides opportunities for sophisticated signal processing and simultaneous reception of multiple beams, which are absent in both analog and subarray digital topologies [2,3,4,5,6,7,8]. Directional and fast-scanning characteristics render phased arrays an attractive approach for various applications, including field surveillance, wireless communication, and electronic warfare [9]. However, the price and occupied volume of beamforming systems limit their ubiquity [10]. From pioneering studies, potential methods of achieving low-cost compact-size element-level digital phased arrays can be classified into three categories: system-on-chip (SoC) integration, advanced packaging, and subarray module miniaturization.

First, advancements in semiconductor technology have enabled the integration of radio frequency (RF) front-end, baseband filtering, and data conversion systems on a single chip at competitive prices and reasonable performance levels [11,12]. Researchers developed a complementary metal-oxide-semiconductor (CMOS) Ku-band transceiver for frequency-modulated continuous-wave (FMCW) radar imaging [13,14], and in another study, they proposed an X-band CMOS four-channel phased array transceiver for synthetic aperture radar (SAR) imaging [15]. Through the use of RF phase-shifting and active switches, an eight-channel silicon-germanium (SiGe) receiver can configure the total number of simultaneous beams for a 2–16 GHz operating frequency [16]. From another perspective, an X-band CMOS FMCW radar transceiver including an on-chip quasi-circulator offers a single-antenna interface to reduce the system form factor [17,18].

Second, advanced packaging methods can enable the integration of active integrated circuits (ICs), passive filtering components, and embedded antennas into a limited space. Under the antenna-in-package scheme, a study presented an X-band active antenna module [19] with a commercial gallium arsenide (GaAs) transceiver, and other studies reported a 64-element phased array module for 5G wireless communication [20]. Another 3-D stacked packaging technique with a thermal-conductive aluminum substrate was developed for producing a high-power X-band T/R module and achieved a compact size of 20 × 20 × 3.7 ${\mathrm{mm}}^{3}$ [21].

Finally, because of the repetitive arrangement in phased array systems, subarray module miniaturization is a good approach for reducing the overall occupied volume. Although the dimensions of an antenna are fundamentally associated with operating frequency and available bandwidth, the use of embedded planar antennas in the tile array configuration can reduce the system form factor [22,23,24,25]. For example, studies have reported that incorporating a SiGe transceiver, a CMOS data converter, and a commercial digital processor can produce a compact subarray module tile for a digital beamforming system [26,27,28]. From another perspective, a study reported a Ku-band multiple-input multiple-output FMCW radar that enables a reduction in the total number of installed antennas through virtual array synthesis and demonstrates high-resolution 3-D imaging capability [29].

Studying essential considerations for realizing phased array hardware can facilitate the development of a low-cost compact-size digital beamforming system; additionally, the design methodology for signal processing back-end systems can be explored. Although element-level digitization enables the development of software-defined phased array radars, extremely high input/output (I/O) bandwidths are occupied by digitized T/R waveforms; consequently, powerful signal processors are required for real-time computing. Benefiting considerably from the shrinking CMOS technology node, field-programmable gate arrays (FPGAs) are equipped with excellent performance per watt, high-speed interfaces, and parallel computing capabilities at reasonable costs and time to market [30,31]. With features of low latency and reduced data throughput, a hierarchical digital beamforming topology [2,9,10,32] has been implemented in FPGAs and employed for real-time radar imaging.

With element-level digitization, digital beamformers can calibrate deterministic phase errors due to unbalanced routing length but still leave random phase fluctuations derived from the system-level clock distribution network. To further improve overall sidelobe rejection, we propose a novel periodic pulse injection circuit embedded in the CMOS transceiver to solve random phase fluctuations. Using this technique, we accomplished clock synchronization with non-overlapping frequency in our radar system using only two RF reference signals. This paper presents the design and implementation of an X-band element-level digital phased array radar utilizing fully integrated CMOS transceivers. We co-designed the radar system and the full-custom CMOS transceiver chips to optimize overall imaging performance. An 8–10 GHz transceiver SoC fabricated in 65 nm CMOS technology offers baseband filtering, frequency translation, and global clock synchronization through the proposed periodic pulse injection technique. Through CMOS SoC integration, antenna-in-package design, and tile array configuration, phased array systems can be miniaturized, and the reduced form factor can bypass volume limitations for various scenarios. The implemented 16-element subarray module with a 1 × 16 configuration accomplishes digital beamforming, back-end computing, and dc–dc conversion with a 317 × 149 × 74.6 mm^3^ size. The subarray modules provide scalability to create a large-scale software-defined beam-steering system through the suggested hierarchical back-end topology, which supports a maximum of 256 radiating elements in the current design.

This paper is an extension of a conference paper that has been accepted [33]. In this paper, beyond the circuit implementation of a fully integrated CMOS transceiver, an additional 16-element radar demonstrator with board-level-integrated front-end amplifiers is proposed, and the results of implementation and verification are presented. The rest of this paper is organized as follows. Section 2 describes the system architecture of the proposed digital phased array radar. Section 3 presents the detailed design methodology of the X-band CMOS transceiver. System integration of the suggested digital phased array radar is explained in Section 4. The experimental results of the CMOS transceiver SoC and radar demonstrator are reported in Section 5, including a performance comparison with other state-of-the-art works. Finally, the study conclusion is provided in Section 6.

## 2. System Architecture of the Proposed Radar

**Figure 1:**
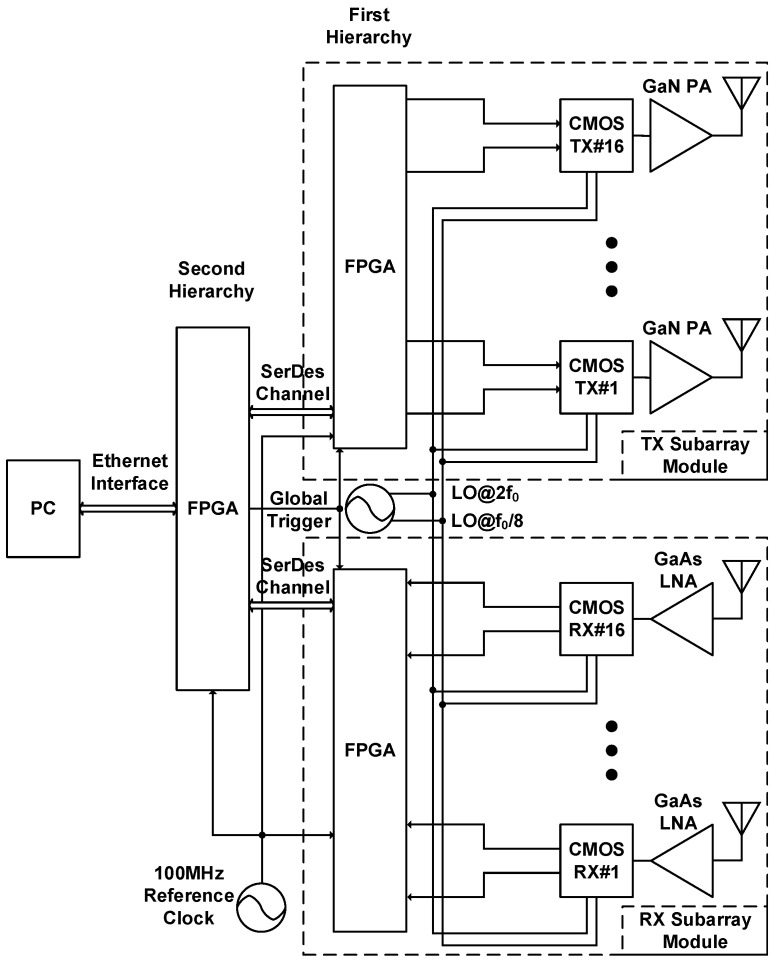
System architecture of the proposed digital phased array radar demonstrator.

Figure 1 depicts the system architecture of the proposed element-level digital phased array radar. Consisting of two 16-element subarray modules and a hierarchical digital back-end, the radar demonstrator generates constructive interference at expected beam-steering directions and achieves range sensing under pulsed radar configuration. One of the subarray modules is operated in transmitting (TX) mode, and the other is operated in receiving (RX) mode. The TX subarray module comprises 16 linear-polarized patch antennas, 16 off-the-shelf front-end GaN power amplifiers, and 16 CMOS transceiver chips in a QFN package. The RX subarray module comprises 16 linear-polarized patch antennas, 16 commercial GaAs low-noise amplifiers, and 16 CMOS transceiver chips in a QFN package. The Qorvo TGA2598-SM GaN driver amplifier [34] and TGA2512-SM GaAs low-noise amplifier [35] are integrated into the RF signal path for the transmitting and receiving subarray module, respectively. Baseband filtering, frequency translation, and global clock synchronization are provided by 8–10 GHz transceiver SoCs through the proposed periodic pulse injection technique. In the transmitting (TX) subarray module, 16 CMOS transceiver chips are only operated in transmitting mode; in the receiving (RX) subarray module, 16 CMOS transceiver chips are only operated in receiving mode. On-chip 10-bit 100 MS/s analog-to-digital converters (ADCs) and 10-bit 100 MS/s digital-to-analog converters (DACs) are responsible for the quadrature-phased baseband signal digitization. The first-hierarchy FPGA is responsible for T/R module control, transmitted waveform excitation, received data acquisition, and digital signal processing (DSP). Digital beamformers implemented in first-hierarchy FPGAs collect relative magnitude and phase information from each array element and calculate essential complex-valued coefficients for the desired beamforming orientation. The second-hierarchy FPGA communicates with the computer through Ethernet and manages data transfer across the array hierarchy over SerDes interfaces. The Analog Devices’ EVAL-ADF5355 PLL evaluation kit [36] is used to generate the necessary local oscillator (LO) reference signals at frequencies of $f_{0}/8$ and $2f_{0}$; $f_{0}$ represents the carrier frequency of each array element and was set to 8.5 GHz in this study for the proposed demonstrator. Moreover, FPGAs administer global timing regulation through a 100 MHz global clock and a global trigger. Four reference signals are distributed from the module input to each array element using active clock trees; system-level synchronization can be realized through these reference signals.

## 3. Circuit Implementation of X-Band Transceiver

**Figure 2:**
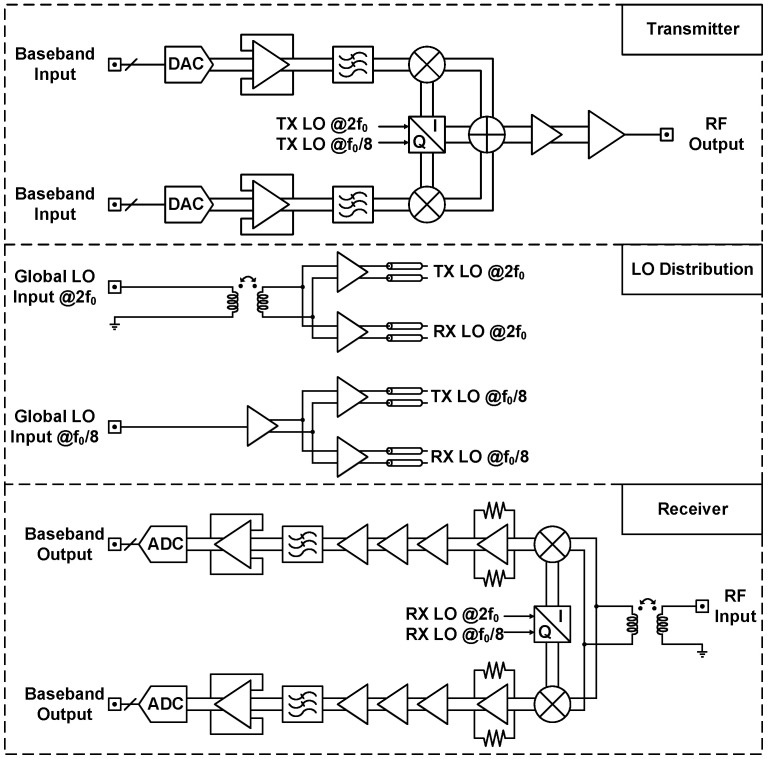
Simplified block diagram of the X-band CMOS transceiver.

The proposed X-band CMOS transceiver serves as a hardware interface between the radiating antenna and back-end digital processor and provides analog signal processing and baseband-to-RF frequency translation. This section describes the circuit design and implementation of the transceiver SoC. We designed and simulated the CMOS circuits with the help of Cadence Virtuoso Analog Design Environment. Figure 2 illustrates a simplified block diagram of the CMOS transceiver circuitry, including the transmitter, receiver, local oscillator (LO) distribution, and quadrature clock generation. Packaging procedures for the reported CMOS chips are introduced in Section 4.1, and experimental results are summarized in Section 5.

### 3.1. Transmitter Design

**Figure 3:**
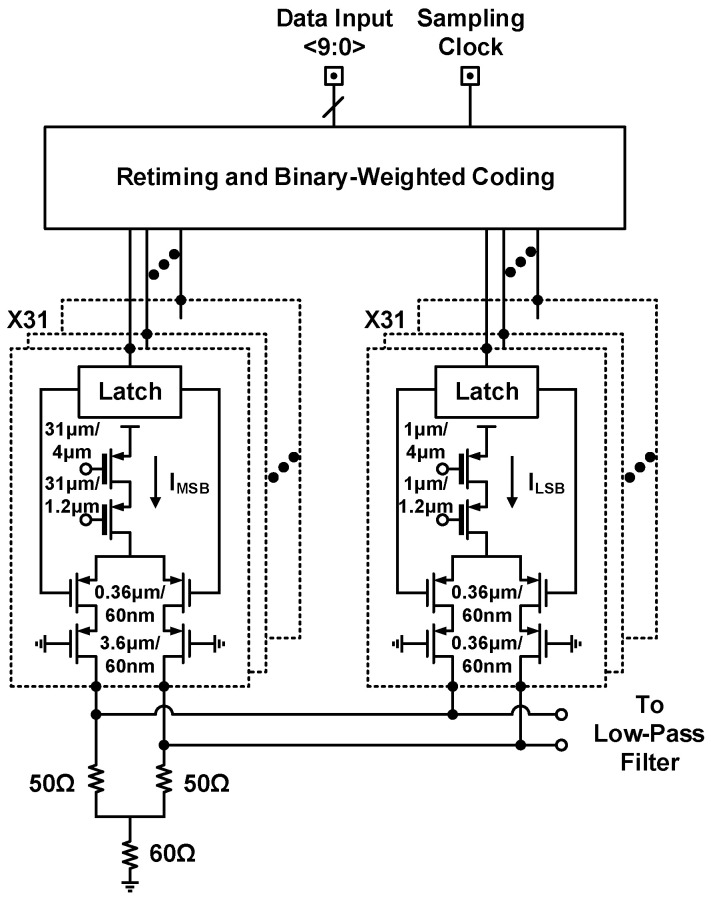
Circuit schematic of the digital-to-analog converter.

As depicted in Figure 2, the implemented transmitter comprises baseband circuitry, a quadrature clock generator, a single-sideband (SSB) mixer, and a power amplifier. Off-chip signal processors deliver digitized data to on-chip 10-bit 100 MS/s current-steering DACs for in-phase and quadrature channels, the circuit schematic of which is presented in Figure 3. Incoming quadrature baseband signals undergo analog filtering and single-sideband modulation through the second-order low-pass filter and the SSB mixer. Subsequently, the power amplifier drives upconverted single-sideband signals to the external 50 Ω load. Quadrature clock generation circuitry executes frequency division and provides orthogonal LO signals at a frequency of $f_{0}$ to mixer switching pairs through $2f_{0}$ and $f_{0}/8$ reference signals; $f_{0}$ denotes the carrier frequency of each transceiver SoC and ranges from 8 to 10 GHz. The design process of the clock generator is reported in Section 3.3.

**Figure 4:**
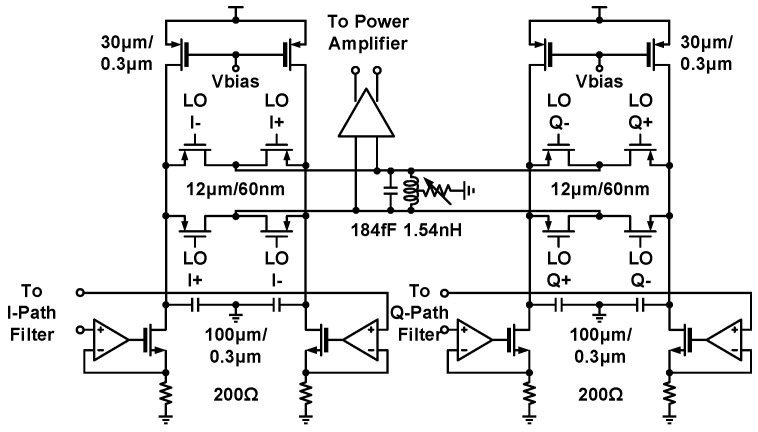
Circuit schematic of the single-sideband mixer.

The complete circuit schematic of an active single-sideband mixer is introduced in Figure 4. Differential baseband signals derived from the preceding low-pass filter are commuted in the form of current by the operational transconductance amplifier; a resistive source degeneration technique is employed to improve amplifier linearity, and shunt capacitors at the output nodes suppress LO feedthrough caused by mixer switching. Two current-driven double-balanced mixer pairs upconvert incoming baseband signals and achieve single-sideband modulation through the combination of quadrature RF currents; a resonant tank is applied to absorb the capacitive load of subsequent circuits. The subsequent power amplifier is driven by the current-mode logic (CML) buffer for SSB mixer differential outputs.

**Figure 5:**
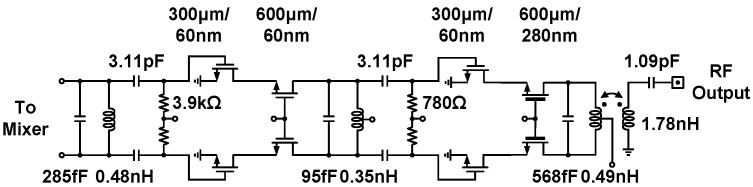
Circuit schematic of the class-AB power amplifier.

The integrated class-AB power amplifier boosts the single-sideband signal and delivers necessary RF power to the off-chip 50 Ω load, the circuit schematic of which is presented in Figure 5. A cascaded two-stage topology [37] that consists of a driver stage and power stage is adopted in the power amplifier design. Both the driver stage and power stage are implemented in the cascode common-source configuration to strengthen the available voltage swings associated with transistor breakdown voltage. The input matching network and the intermediate matching network apply resonant circuits to suppress out-of-band aggressors and absorb the transistor parasitic capacitors with compact areas. The power stage amplifier tolerates much higher voltage swing through the use of thick-gate-oxide common-gate devices and a 2.5 V drain bias voltage; subsequently, the transformer-based output network executes power combination and impedance conversion. The implemented power amplifier was determined to achieve a simulated drain efficiency of 18% and an output power of 18.3 dBm at 10 GHz.

### 3.2. Receiver Design

As shown in Figure 2, the implemented direct-conversion quadrature receiver comprises a mixer-first RF front-end, a quadrature clock generator, and baseband circuitry. The incoming RF input signal undergoes frequency translation and analog signal processing; subsequently, the downconverted quadrature baseband signals are distributed to the off-chip signal processor through the digital interface. Both the transmitter and receiver are equipped with separate quadrature clock generation circuits to perform frequency division. The design is described in detail in Section 3.3.

**Figure 6:**
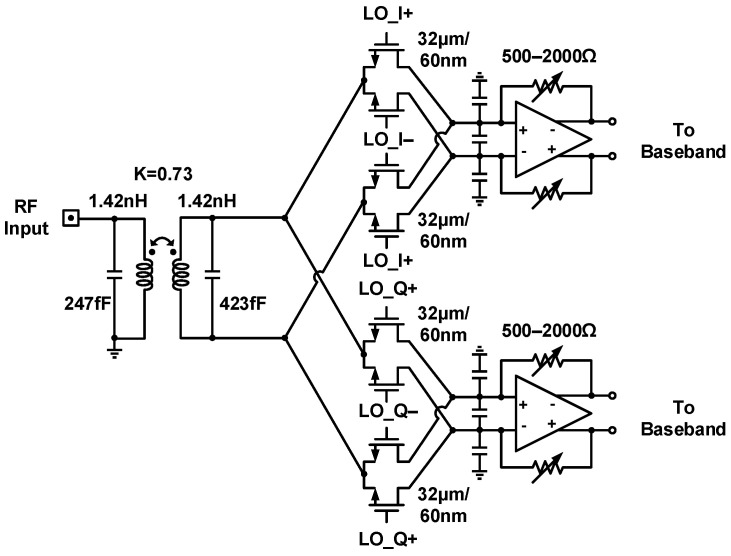
Circuit schematic of the mixer-first receiver front-end.

The circuit schematic of the mixer-first RF front-end is illustrated in Figure 6. A transformer-based input matching network is adopted for single-ended-to-differential (S2D) conversion and dc current isolation. Current-driven passive mixers downconvert received RF signals and can tolerate simultaneous transmitter leakage. The transimpedance amplifiers (TIAs) provide current-to-voltage conversion for downconverted waveforms and drive subsequent baseband circuitry activity. However, in the absence of a low-noise amplifier, the input impedance of the TIAs is upconverted by a bidirectional passive mixer and can be directly observed at the receiver RF input port. Analyses of the impedance transparency feature in a mixer-first architecture are presented in [38,39]. Through the reported analysis procedures, the design of impedance matching and baseband load entails making tradeoffs among conversion gain, noise figure, and mixer LO driver power dissipation to achieve the desired 8–10 GHz operating frequency.

**Figure 7:**
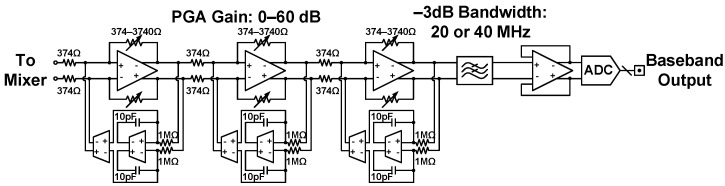
Circuit schematic of the baseband circuitry.

**Figure 8:**
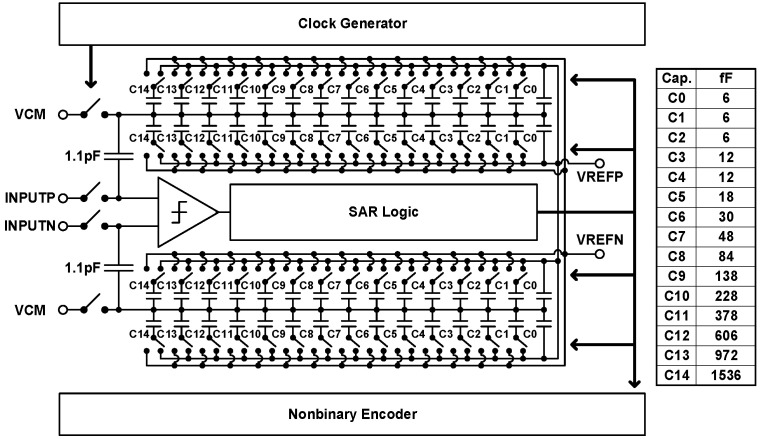
Circuit schematic of the analog-to-digital converter.

Figure 7 depicts the baseband circuitry [18]. Downconverted received signals undergo amplification and filtering through the programmable gain amplifier (PGA) and low-pass filter. With dc offset cancellation and feedback resistor control, a three-stage PGA accomplishes a simulated 60 dB gain tuning range and 1 dB gain tuning step, followed by a second-order multiple feedback low-pass filter with digital-controlled reconfigurable feedback resistors and capacitors. The overall filtering bandwidth is either 20 MHz or 40 MHz. Off-chip signal processors use sampled data from on-chip 10-bit 100 MS/s nonbinary redundant successive approximation register analog-to-digital converters (SAR ADCs) for in-phase and quadrature channels, the circuit schematic of which is presented in Figure 8.

### 3.3. LO Distribution and Quadrature Clock Generation

In this study, two synchronized external LO signals at frequencies of $2f_{0}$ and $f_{0}/8$ were delivered to both the transmitter and receiver equipped with quadrature clock generation circuitry; $f_{0}$ denotes the carrier frequency of each transceiver SoC and ranges from 8 to 10 GHz. Clock generation circuits execute frequency division and provide orthogonal LO signals at a frequency of $f_{0}$ to the in-phase and quadrature (I/Q) mixers. Element-level digitization can calibrate deterministic phase errors while leaving random uncertainties, which are partially derived from temperature-induced phase fluctuations in the active LO distribution network and 180${}^{\circ }$ phase uncertainty in the frequency division process. These problems can be solved by applying bandgap reference voltage biasing and the proposed periodic pulse injection technique. Detailed design processes are described as follows.

**Figure 9:**
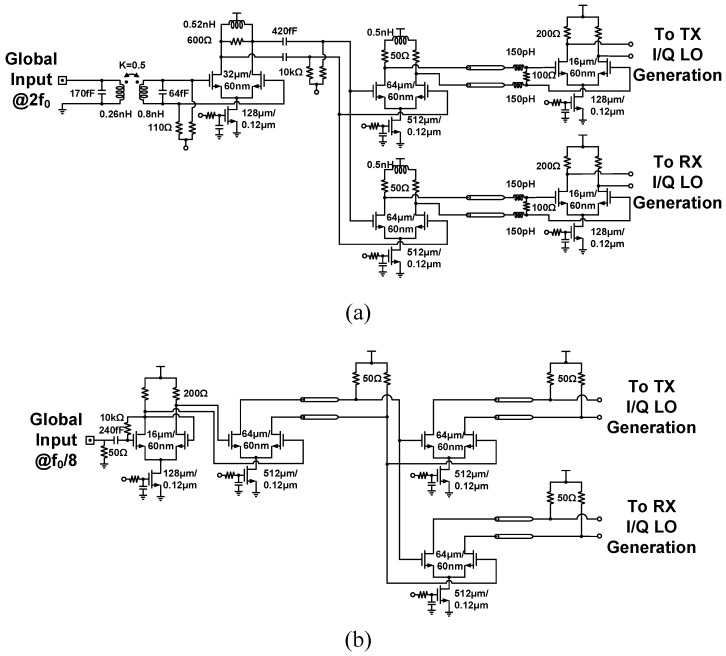
Circuit schematic of the clock distribution network composed of (**a**) $2f_{0}$ LO buffer chain and (**b**) $f_{0}/8$ LO buffer chain.

**Figure 10:**
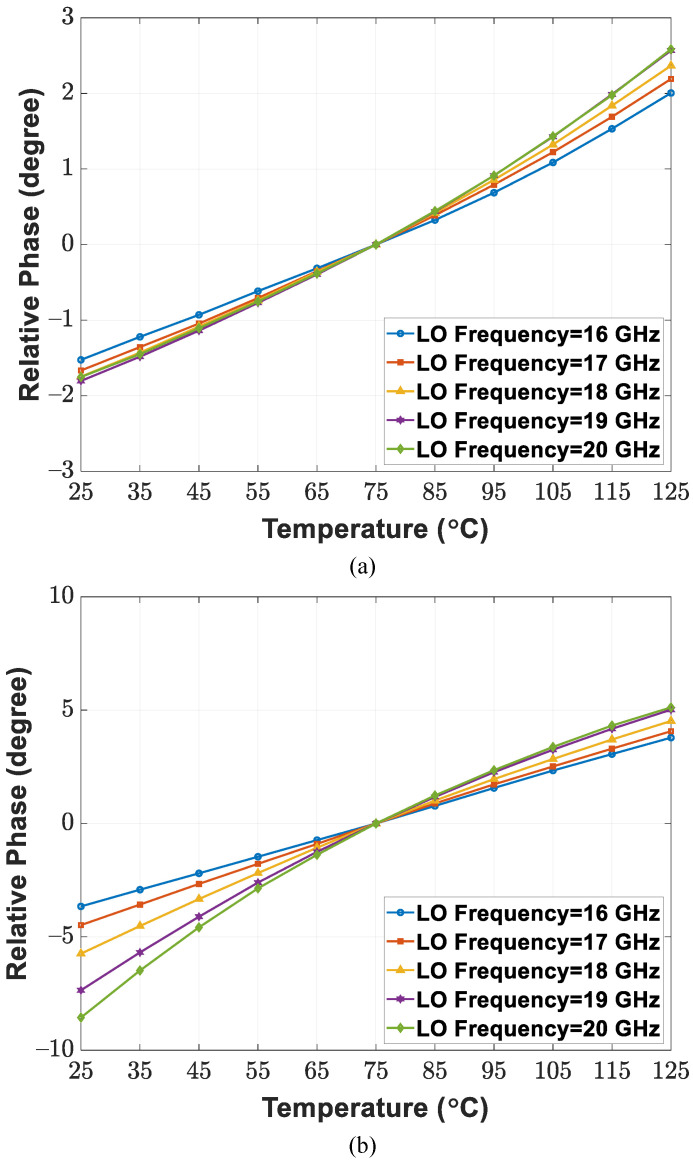
Simulated relationship between the relative phase of LO signal and environment temperature under (**a**) activating and (**b**) deactivating bandgap reference voltage biasing.

Figure 9a,b depict the circuit implementation of the LO distribution network composed of a $2f_{0}$ buffer chain and $f_{0}/8$ buffer chain. An on-chip bandgap reference circuit provides temperature-insensitive biasing voltages to CML buffers. Moreover, an inductive gain-peaking technique [40] is also introduced to compensate for power loss derived from signal routings. Conversely, with a relatively low frequency, the $f_{0}/8$ buffer chain applies a CML-based active S2D circuit and 50 Ω load CML buffers directly. Figure 10a,b present the simulated relationship between the relative phase and environment temperature for 16–20 GHz LO signals. In the 25–125 ${}^{\circ }$C operating temperature range, the CML buffer chain exhibits 13.7${}^{\circ }$ and 4.4${}^{\circ }$ maximum phase deviations under activating and deactivating bandgap reference biasing configurations, respectively. Applying bandgap reference biasing to CML buffers is confirmed to reduce temperature-induced phase fluctuation in the LO path.

**Figure 11:**
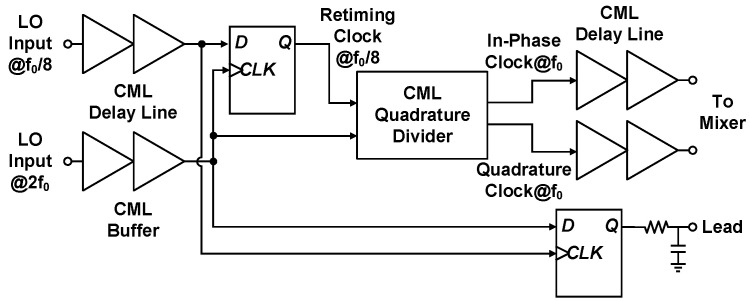
Simplified block diagram of the proposed quadrature clock generation circuit.

The $2f_{0}$ fed signal, along with the frequency division process, prevents substantial LO leakage at transceiver RF ports. However, the divide-by-2 process introduces 180${}^{\circ }$ phase uncertainty to quadrature clock signals and causes difficulty in array element synchronization; that is, the output signals of the two synchronized CMOS SoCs may be either in phase or out of phase once the CMOS transceivers restart. Consequently, beamforming fails to operate because of arbitrary constructive or destructive interference. To solve the 180${}^{\circ }$ phase ambiguity, our study proposes a quadrature clock generation circuit with a periodic pulse injection technique. A simplified block diagram is presented in Figure 11, where signals are presented in a single-ended form for clarity. A CML quadrature divider accomplishes frequency division and orthogonal clock generation from the buffered $2f_{0}$ signal delivered by the LO distribution network. Programmable CML delay lines drive either the transmitter or receiver mixers and enable I/Q phase imbalance calibration by altering the relative delay between I/Q clocks. Similarly, the CML circuits employ proportional-to-absolute-temperature biasing voltages to avoid temperature-induced phase fluctuation in the LO path.

**Figure 12:**
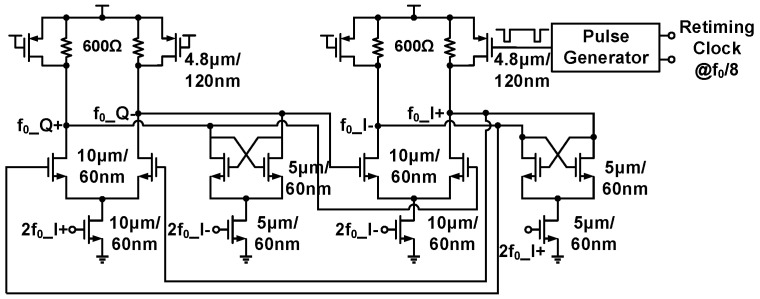
Circuit schematic of the CML quadrature divider with periodic pulse injection.

**Figure 13:**
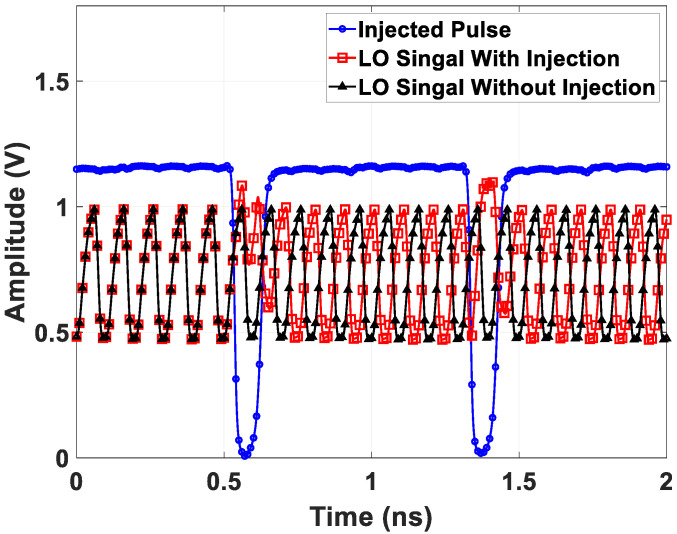
Simulated 10 GHz LO waveforms under deactivating and activating 1.25 GHz pulse injection.

An additional $f_{0}/8$ path is introduced to eliminate phase uncertainty. Initially, the buffered $f_{0}/8$ signal undergoes a retiming process by the $2f_{0}$ signal for clock edge alignment. Subsequently, a periodic pulse signal with an adjustable duty cycle and delay is produced by the pulse generator circuit from the $f_{0}/8$ retiming clocks and is fed into the CML quadrature divider, as depicted in Figure 12. Finally, the pulse signal turns on a pMOS device and injects current into the divider output node periodically. The injected current increases the output voltage, consequently affecting the absolute phase of the quadrature signals, depending on the selected injection point. Simulated 10 GHz LO waveforms along with a 1.25 GHz pulse signal are depicted in Figure 13. Comparing the activating injecting case with the deactivating injecting case reveals a 180${}^{\circ }$ phase difference after the pulse signal triggers the divider circuitry. Thus, the proposed quadrature divider can regulate absolute phases of output clocks through a chosen injection point (i.e., the relative delay between the $f_{0}/8$ retiming clock and generated pulse), and a well-defined relative phase relationship between any two transceiver chips is achievable. Furthermore, to prevent metastability in the retiming process, the proposed quadrature clock generation circuitry adopts an additional path to monitor the clock edge relationship between the $2f_{0}$ and $f_{0}/8$ signals.

## 4. System Integration of the Proposed Radar

This section describes the system integration of the proposed element-level digital phased array radar, which incorporates radiating element packaging and subarray module assembly. The corresponding experimental results are summarized in Section 5.

### 4.1. Array Element Packaging

An array element is the basic functional unit in a beamforming system and is responsible for transmitting or receiving expected signals through an embedded antenna. Each array element comprises a T/R module and the corresponding part of the interposer board on which the T/R module is mounted.

Our previous work [33] reported the antenna-in-package (AiP) method for T/R module packaging. The integration of the compact AiP module and high-performance front-end amplifiers can further improve the radiation power and noise figure at the cost of increased heat density. For example, the expected power density of the Qorvo TGA2598-SM GaN driver amplifier [34] is 0.48 W$/{\mathrm{mm}}^{2}$. Because the top layer is occupied by a patch antenna and the bottom layer is epoxy encapsulated, external heat sinks are unavailable on the surface of the AiP module [33]; consequently, the heat generated by the active components spreads to the poor-thermal-conductivity ceramic substrate and the interposer board through solder balls. Additional off-chip front-end amplifiers increase the difficulty of heat dissipation in an ultrathin tile array. An alternative method for board-level integration of the CMOS T/R quad flat no-lead (QFN) module, off-the-shelf front-end amplifier, and linear-polarized patch antenna was adopted in this study and is explained as follows.

**Figure 14:**
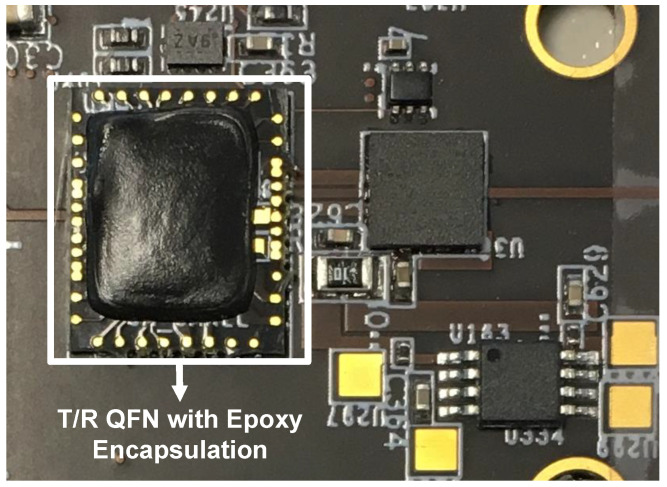
Top view of a CMOS T/R QFN module.

**Figure 15:**
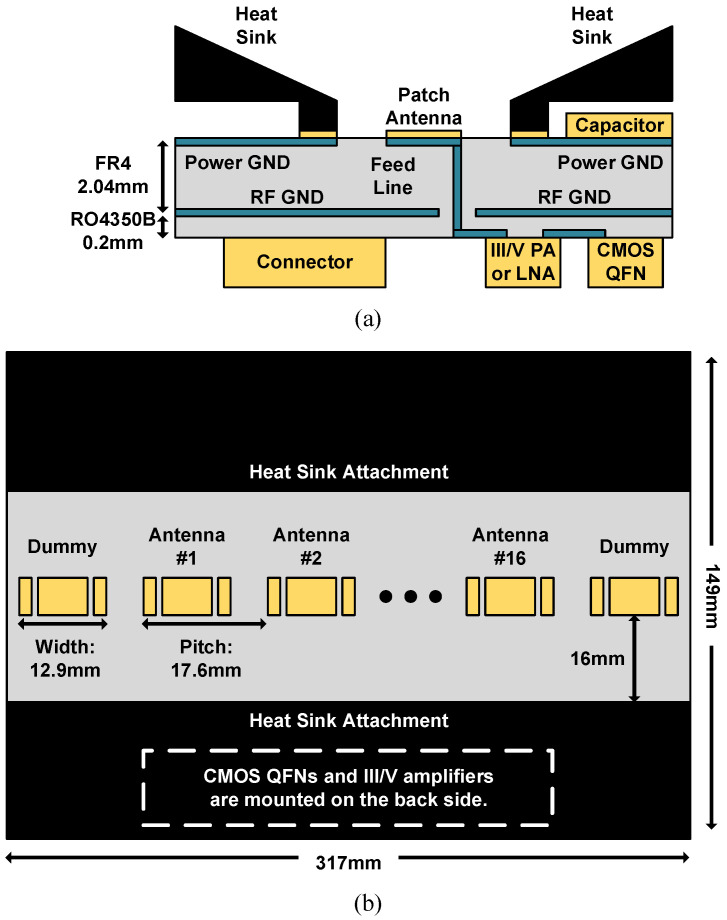
(**a**) Side view and (**b**) top view of a 1 × 16 interposer board.

To ensure expedited shipping and preliminary verification processes on a limited budget, only an FR4 substrate was chosen for the QFN module and the front-end interposer board. As shown in Figure 14, the T/R QFN module includes a wire-bonded CMOS transceiver SoC and surrounding filtering capacitors; power rails, digital interfaces, baseband analog signals, and RF LO signals come into contact with the interposer board through bonding pads. On-chip 10-bit 100 MS/s ADCs and 10-bit 100 MS/s DACs are responsible for the baseband signal digitization. Under epoxy encapsulation, a packaged QFN module is 9.35 × 7.75 × 1.39 ${\mathrm{mm}}^{3}$ in size. Figure 15a,b depict the side view and top view of a 1 × 16 front-end interposer board including 16 array elements. Each array element is composed of the T/R QFN module, commercial front-end amplifier, and redesigned linear-polarized patch antenna. The edge-to-edge dimension of embedded patch antennas is set to 17.6 mm to avoid a grating lobe at 8.5 GHz operating frequency. The ground layer in the middle of the FR4 substrate serves as an antenna ground and provides electromagnetic interference shielding for RF signals. Due to limited resources, only one-layer Rogers RO4350B is embedded into the front-end interposer board to improve insertion loss between the commercial front-end amplifier and the patch antenna.

**Figure 16:**
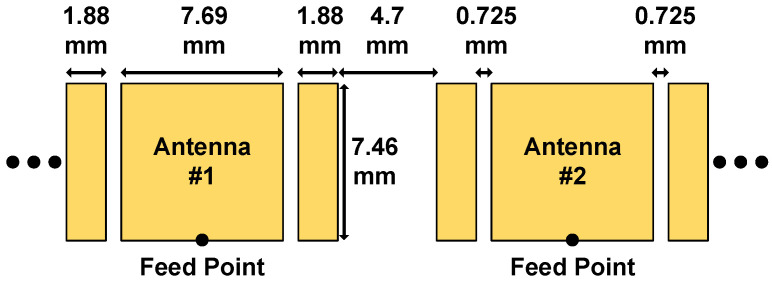
Dimensions for the patch antenna.

**Figure 17:**
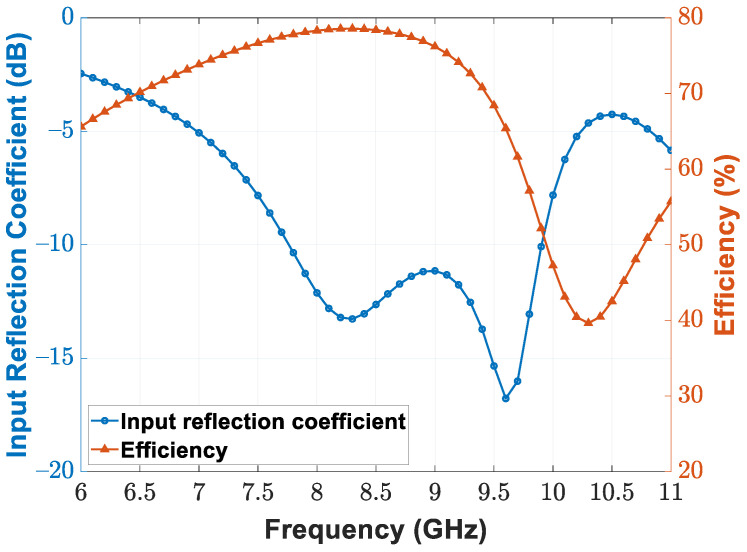
Simulated input reflection coefficient and radiation efficiency of the patch antenna.

**Figure 18:**
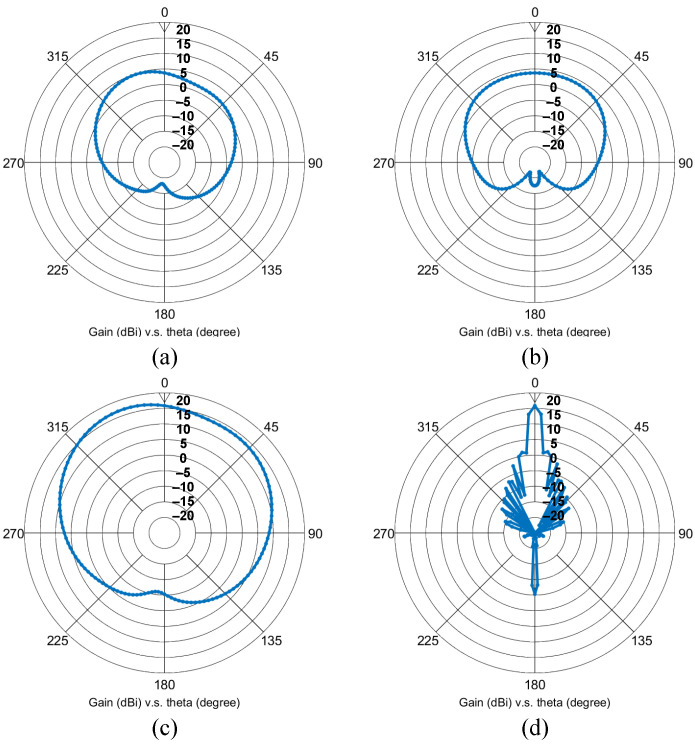
Simulated (**a**) E-plane and (**b**) H-plane radiation pattern for the single patch antenna; simulated (**c**) E-plane and (**d**) H-plane radiation pattern for the 16-element patch antenna array.

Detailed antenna dimensions are presented in Figure 16. Moreover, Figure 17 depicts the simulated input reflection coefficient and radiation efficiency of the patch antenna incorporating a vertical via and horizontal microstrip feed line. We designed and simulated the antenna with the help of Keysight Advanced Design System (ADS). Occupying a 7.8–9.9 GHz bandwidth, the implemented patch antenna achieves 78.4% and 52.2% radiation efficiency at the 8.5 GHz and 9.9 GHz operating frequencies, respectively. Figure 18a,b illustrate the far-field cut of the simulated radiation pattern for the E-plane and H-plane, respectively. Equipped with a maximum antenna gain of 4.741 dBi and 3.737 dBi for the E-plane and H-plane, the patch antenna accomplishes a half-power beamwidth (HPBW) of $78^{\circ }$ for the E-plane and an HPBW of $120^{\circ }$ for the H-plane. Furthermore, we simulated the radiation pattern of the implemented patch antenna array and present the simulation results of the 16-element antenna array at the 8.5 GHz center frequency. Figure 18c,d depict the simulated E-plane and H-plane radiation patterns for the 16-element antenna array under rectangular window extraction and $0^{\circ }$ beamforming angle. For the E-plane cut, the 16-element antenna array achieved a maximum gain of 16.873 dBi and HPBW of $78^{\circ }$; for the H-plane cut, that is to say, our desired beamforming direction, the 16-element antenna array achieved a maximum gain of 15.879 dBi, HPBW of $6^{\circ }$, a peak sidelobe ratio of −14.52 dB, and a 12.142 dB gain improvement due to beamforming.

### 4.2. Assembly of 1 × 16 Subarray Module

**Figure 19:**
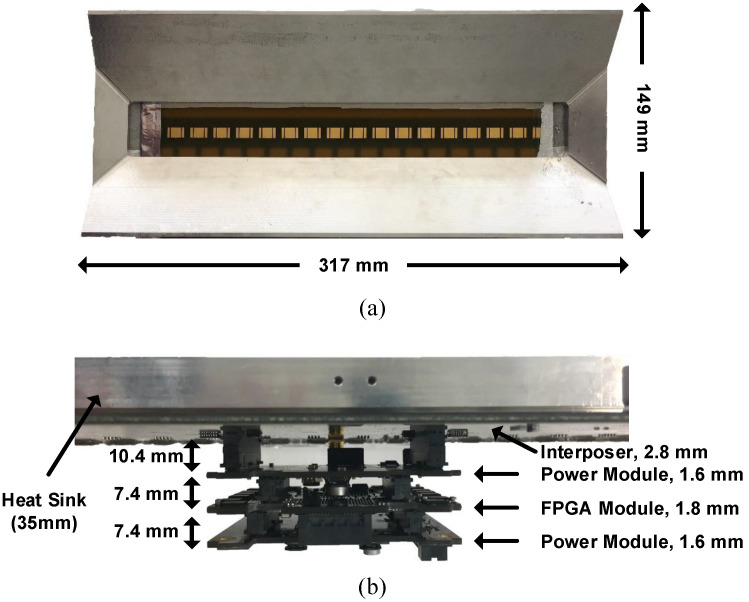
(**a**) Top view and (**b**) side view of a 1 × 16 subarray module.

Figure 19a,b depict the implemented 1 × 16 subarray module. The 1 × 16 subarray module comprises a front-end interposer board, a signal processor board, and two power module boards. In contrast to the 4 × 4 configuration [33], the 1 × 16 system replaces the T/R AiP module with the combination of the T/R QFN module, off-the-shelf front-end amplifier, and redesigned patch antenna for each radiating element. Sixteen equally spaced radiating elements execute baseband-to-RF frequency translation and produce constructive interference at the expected beam-steering directions. The signal processor, Xilinx FPGA SoC, communicates with radiating elements through physical routings in the interposer board and executes cross-hierarchy data transfer over the SerDes interface. Moreover, as mentioned in Section 3.3, each CMOS transceiver requires two external LO signals at frequencies of $f_{0}/8$ and $2f_{0}$ to eliminate phase uncertainty and generate quadrature clocks; the FPGAs also administer global timing regulation through a 100 MHz global clock and a global trigger. Four reference signals are distributed from the module input to each array element using active clock trees; system-level synchronization can be realized through these reference signals. Additionally, two customized power module boards generate essential supply voltages from a single 28 V dc input rail with high-efficiency dc–dc conversion. In our current implementation, the 1 × 16 subarray module achieves a size of 317 × 149 × 74.6 ${\mathrm{mm}}^{3}$, including a heat sink mounted on the interposer board for convection cooling. An improved thermal management system incorporating heat pipes and heat sinks may help further reduce the module volume.

## 5. Experimental Results

This section presents experimental results, including the circuit performance of the X-band CMOS transceiver and functional verification of the 1 × 16 subarray modules, for the proposed element-level digital phased array radar. After fabricating the transceiver SoCs, wafer-level measurements were performed to evaluate circuit specifications, and then qualified chips were packaged according to the assembly procedures detailed in Section 4. Subsequently, a finished radar demonstrator composed of two 1 × 16 subarray modules was subjected to power-on calibration, antenna pattern measurement, and range sensing experimentation.

### 5.1. CMOS Transceiver

**Figure 20:**
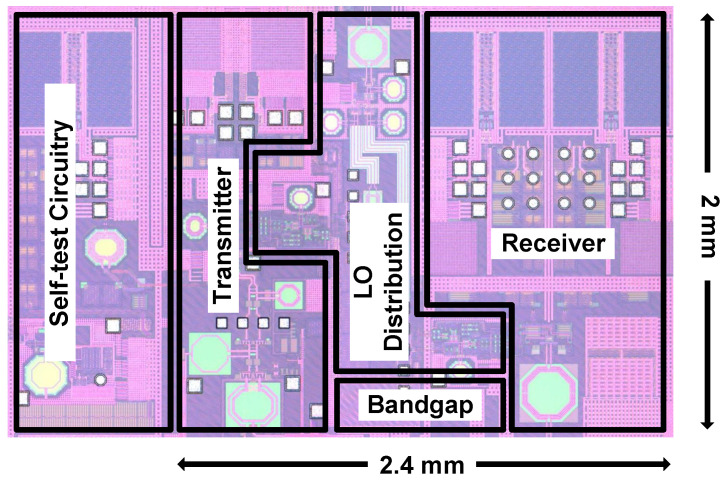
Micrograph of the implemented CMOS transceiver chip.

**Figure 21:**
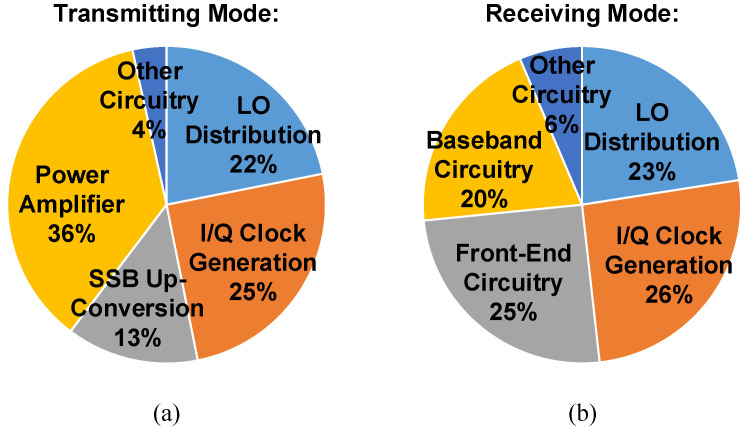
Power consumption of the CMOS transceiver operated in (**a**) transmitting mode and (**b**) receiving mode.

This paper presents a fully integrated X-band transceiver SoC fabricated using 65 nm CMOS technology. Figure 20 presents a micrograph of the implemented chip. Occupying a chip area of 2.4 × 2 ${\mathrm{mm}}^{2}$, the CMOS transceiver consumes a total power of 1.45 W in transmitting mode and 1.41 W in receiving mode on 1.2, 1.8, and 2.5 V dc power rails. The power consumption of key transmitting and receiving blocks is illustrated in Figure 21a,b. The substantial power dissipation is mainly derived from power-hungry CML circuits and on-chip low-dropout regulators (LDOs), which are employed to reduce temperature-induced phase fluctuations and eliminate system-level switching power supply noise, respectively.

**Figure 22:**
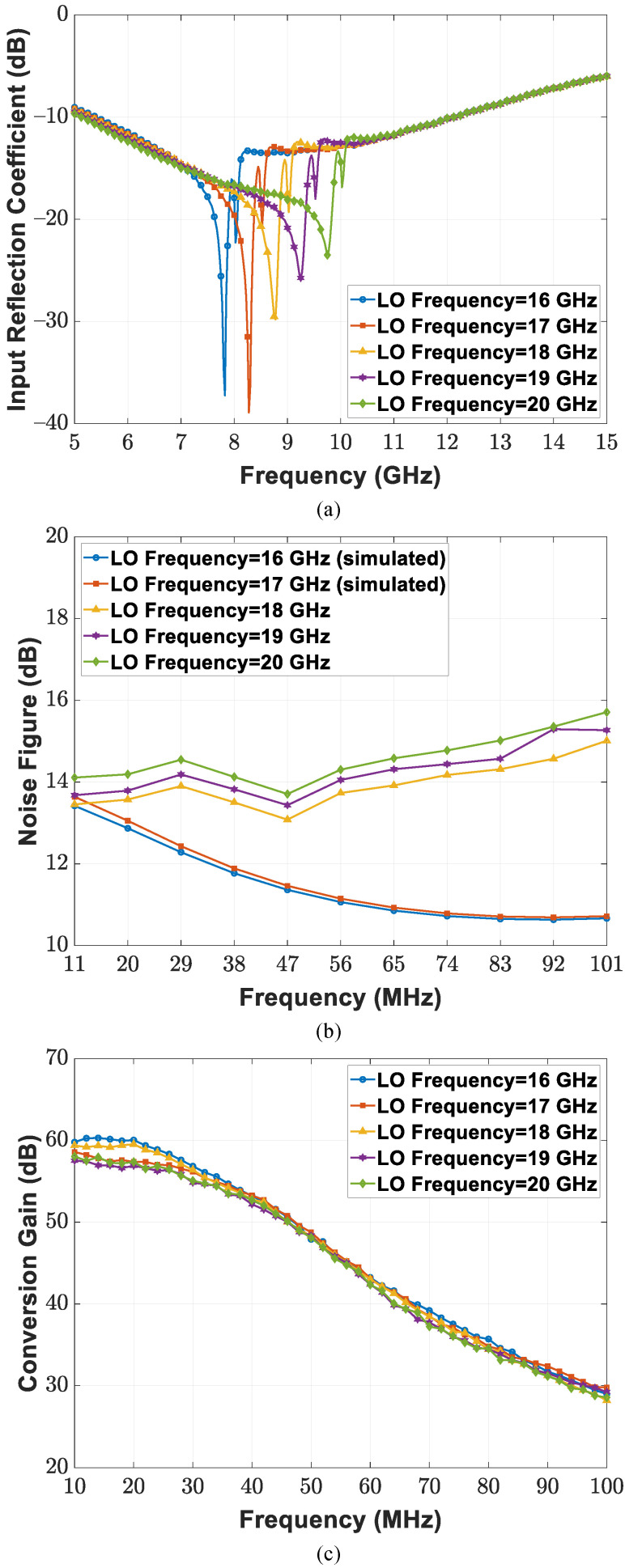
Circuit performance of the implemented receiver, including (**a**) input reflection coefficient, (**b**) noise figure, and (**c**) conversion gain.

**Figure 23:**
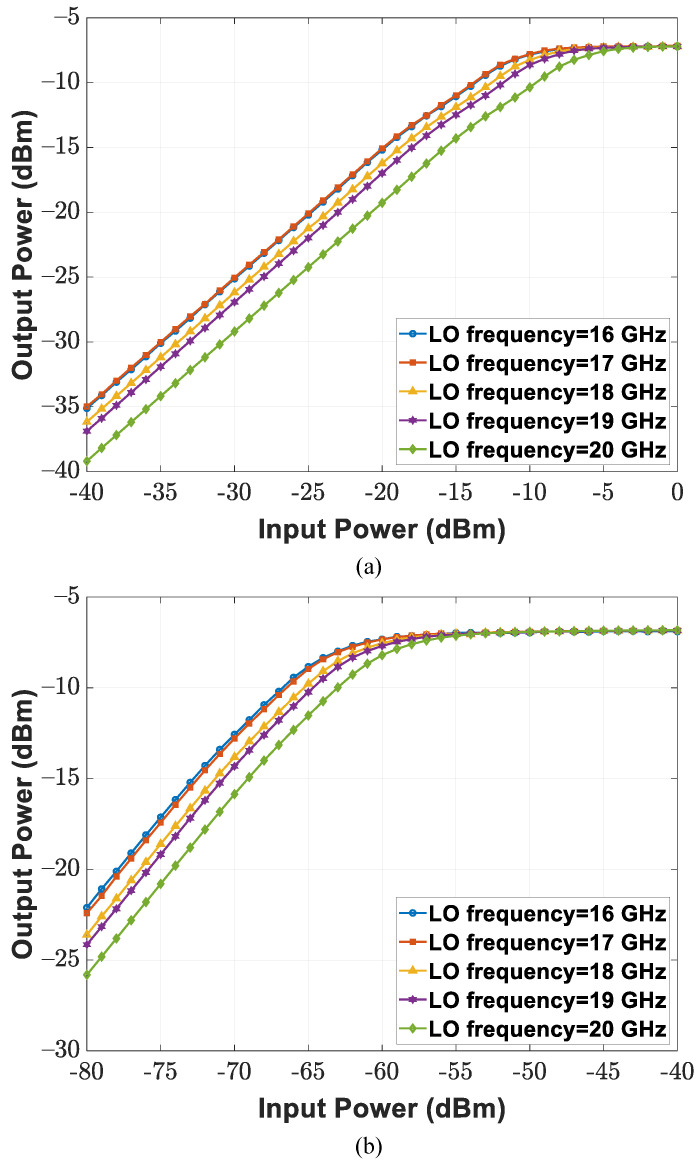
The 1 dB compression point ($P_{1dB}$) of the implemented receiver for (**a**) lowest conversion gain and (**b**) highest conversion gain.

**Figure 24:**
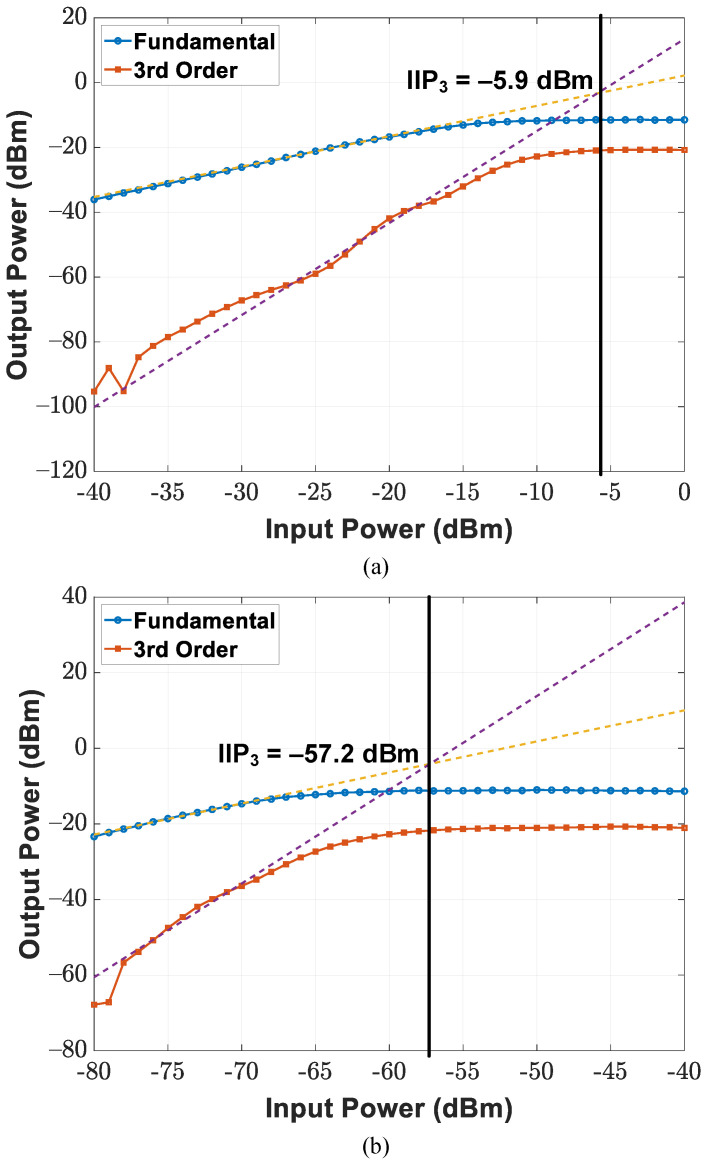
Third-order intercept point ($IP_{3}$) of the implemented receiver for (**a**) lowest conversion gain and (**b**) highest conversion gain.

Circuit measurement specifications of the CMOS transceiver SoC under the wafer probing scheme are summarized as follows. By utilizing the mixer-first RF front-end, the receiver chain suppresses out-of-band interference through narrow-band noise performance and input matching; band switching operation is realized by controlling the reference frequency of the external LO signal. Figure 22a depicts the input reflection coefficient of the implemented receiver with a 50 Ω source impedance at LO frequencies ranging between 16 and 20 GHz; a Keysight E8257D analog signal generator provides −10 dBm RF power at the given LO frequencies. The minimum input reflection coefficient is achievable with a frequency that is near the given carrier frequency (i.e., half of the LO frequency), and the magnitude of the reflection coefficient increases as the measured frequency moves away from the selected operation band. The double-sideband (DSB) noise figure and conversion gain are illustrated in Figure 22b,c. The implemented receiver chain achieves a 14.6 dB DSB noise figure and maximum DSB conversion gain of 57.2 dB within the 40 MHz baseband bandwidth at a 20 GHz LO frequency. For linearity measurements, single-tone tests were performed at an 18 MHz baseband frequency and different LO frequencies. Figure 23a,b display measurement results of the receiver 1 dB compression point ($P_{1dB}$) under the lowest conversion gain of 3.8 dB and highest conversion gain of 57.2 dB. The input $P_{1dB}$ reaches −14 and −66 dBm at an 18 GHz LO frequency for the lowest gain and highest gain cases, respectively. Moreover, two-tone tests were conducted at baseband frequencies of 18 and 20 MHz. The input third-order intercept points ($IIP_{3}$) of the implemented receiver are presented in Figure 24a,b, including those for both the lowest conversion gain and highest conversion gain cases. $IIP_{3}$ reaches −5.9 dBm for the lowest conversion gain case and −57.2 dBm for the highest conversion gain case at the 18 GHz LO frequency.

**Figure 25:**
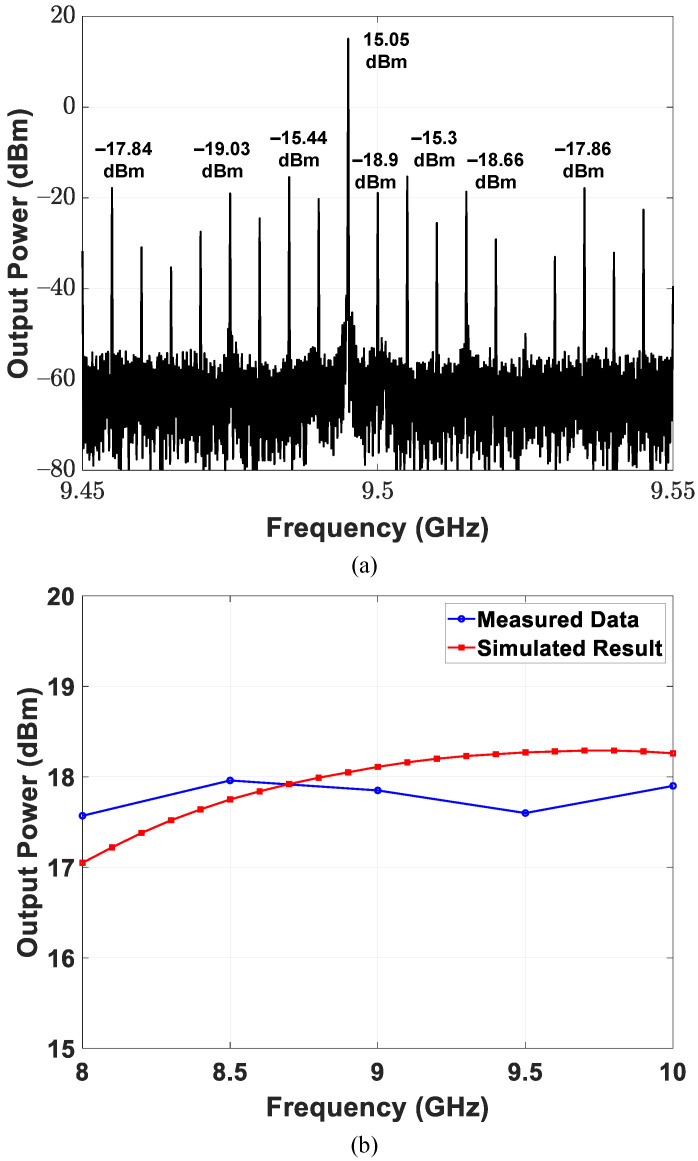
Circuit performance of the implemented transmitter, including (**a**) output spectrum at 19 GHz LO and 5 MHz baseband input frequencies, (**b**) saturated output power.

The performance of the transmitter incorporating a spurious level, carrier leakage, saturated output power, and pulse-mode operation capability was examined. Figure 25a shows the output spectrum of the transmitter at 19 GHz LO and 5 MHz baseband input frequencies; lower-sideband (LSB) upconversion was demonstrated in the experiment. The implemented transmitter achieves a spurious level of −30.35 dBc and a carrier leakage of −33.95 dBc. The measured and simulated saturated output power among 8–10 GHz are presented in Figure 25b. In an 8–10 GHz operating frequency range, the CMOS power amplifier exhibits a peak saturated output power of 17.96 dBm and a peak drain efficiency of 11.9% at 8.5 GHz.

### 5.2. Radar Demonstrator with 1 × 16 Subarray Modules

**Figure 26:**
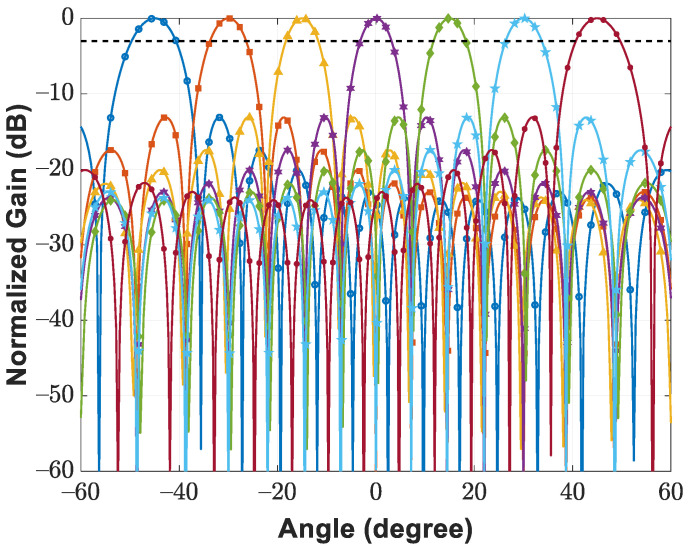
Measured antenna patterns for 16-element demonstrator operated in receiving mode.

This paper presents a 16-element radar demonstrator incorporating a hierarchical digital back-end and two 1 × 16 subarray modules with board-level integrated RF front-end amplifiers. As depicted in Figure 1, one of the 1 × 16 subarray modules is operated in transmitting mode, and the other is operated in receiving mode. Additionally, the module operated in transmitting mode is vertically arranged for elevation recognition, whereas that operating in receiving mode is horizontally configured for azimuth recognition through simultaneous multiangle scanning [41]. Digital beamformers implemented in the first-hierarchy FPGA are responsible for phase shifting and magnitude amplification, depending on the beam-steering orientation. After the successful execution of power-on calibrations [33], the performance of the digital beamformer was evaluated through the verification of antenna patterns. The measured E-plane antenna patterns for the demonstrator operating in receiving mode are presented in Figure 26, with the mainlobe being steered from $-45^{\circ }$ to $45^{\circ }$ at $15^{\circ }$ steps. By applying the kaiser window with a shape factor of 3, this study determined that the digital beamformer achieved an average sidelobe level of −25.64 dB and a half-power beamwidth of $8.06^{\circ }$ at a $0^{\circ }$ beamforming angle; additionally, at the same angle, an average sidelobe level of −13.11 dB and a half-power beamwidth of $6.27^{\circ }$ were achieved without tapering. Benefiting from the digital beamforming scheme, the proposed 16-element demonstrator supports pulsed radar configuration; the corresponding experimental results are reported as follows.

A pulsed radar evaluates the target distance according to the time of flight between the radar-transmitted signal and target-reflected signal. The maximum unambiguous range of the pulsed radar can be expressed as follows. $f_{PRF}$ and *c* represent the pulse repetition frequency and the speed of light, respectively.

(1) $$\mathrm{Maximum}\;\mathrm{unambiguous}\;\mathrm{range}=\frac{c}{2\cdot f_{PRF}}$$

An up-chirp signal with a duration of 320 ns and sweep bandwidth of 20 MHz is employed for the transmitted pulse, and a 7.68 μs pulse repetition period and a 4.17% duty cycle are adopted for the radar demonstrator. Theoretically, a 1152 m maximum unambiguous range and 7.5 m range resolution can be achieved with the current system specifications. Because the transmitter and receiver are not simultaneously activated, the CMOS receiver avoids transmitter-induced baseband saturation and can increase the PGA gain for long-distance target sensing. Initially, each array element delivers digitized echo signals to the digital back-end. The DSP functional block implemented in the first-hierarchy FPGA executes waveform averaging for pulsed radar operation. Subsequently, processed data are delivered to MATLAB through an Ethernet interface. Matched filtering and digital beamforming are realized in MATLAB for time-of-flight calculation and spatial filtering with acceptable latency. Finally, the captured range–azimuth information for the given scene is displayed on the screen.

**Figure 27:**
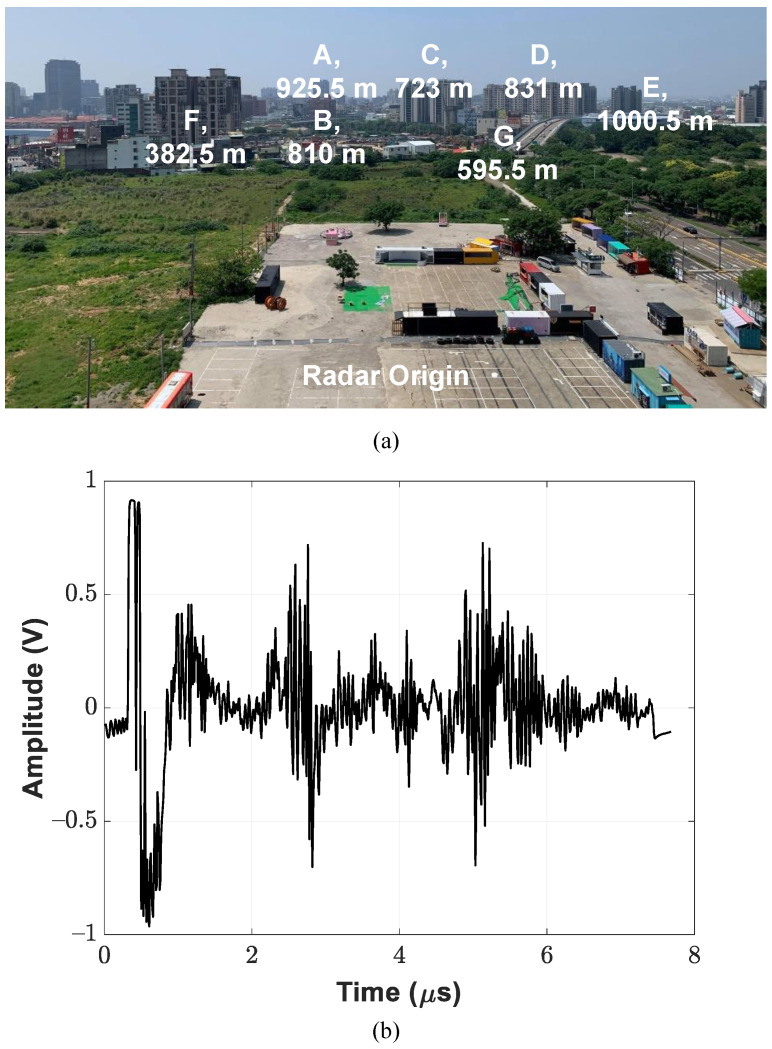
(**a**) Snapshot of target buildings. (**b**) Downconverted received waveform with pulse repetition period of 7.68 μs.

The 16-element radar demonstrator was placed on the seventh floor; the field of view seen by the demonstrator is depicted in Figure 27a, in which the target buildings and corresponding distance are labeled. An experiment was performed for functional verification of the pulsed radar, and the downconverted received waveform for a single pulse repetition period is displayed in Figure 27b. Once the radar demonstrator activates the receiver modules, the dc offset cancellation circuitry implemented in the CMOS PGA chain is triggered to update the bias point through analog feedback loops. During the settling process of the feedback loop, the receiver provides relatively little conversion gain; the blind range of the pulse radar demonstrator is broadened due to this effect.

**Figure 28:**
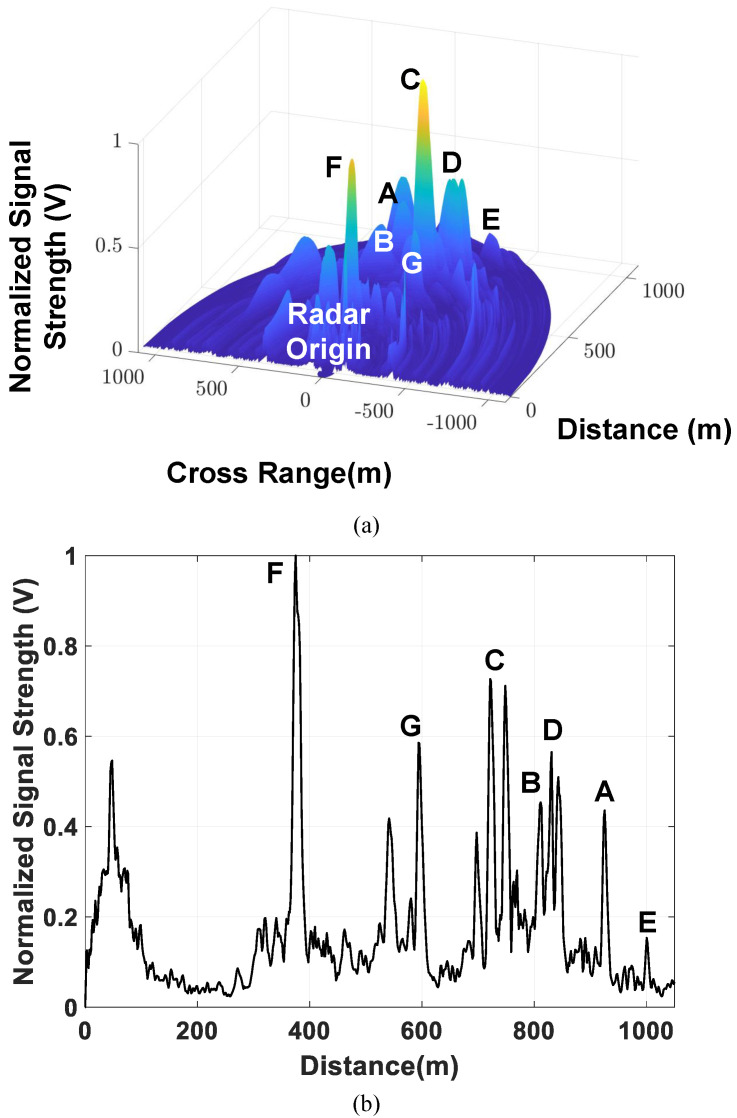
(**a**) Isometric view and (**b**) side view of the captured range–azimuth plot by using 16-element radar demonstrator.

Isometric and side views of the captured range–azimuth plot for labeled target buildings are presented in Figure 28a,b; the colored z-axis in the figure indicates the relative signal magnitude on a linear scale, and its value is normalized to the maximum receiving signal strength.

The target locations evaluated by the proposed radar demonstrator were confirmed to match the labeled buildings illustrated on the map. A 1000.5 m maximum observation range was accomplished for stationary buildings. To generate a complete pulsed radar image, the average latency between the user request and radar image display is 150 ms for the current hardware implementation, consisting of 2 ms for 256 consecutive measurements, 13 ms for data transfer through SerDes and Ethernet interfaces, and 135 ms for data processing in MATLAB. Further acceleration can be achieved by realizing the matched filtering and radar imaging algorithms in FPGAs. A comparison of the performance of the developed radar transceiver with other state-of-the-art works is summarized and reported in Table 1. Although the power consumption is not superior to that reported in prior studies, the performance of the proposed transceiver in terms of the spurious level and available conversion gain is comparable to that achieved in the state-of-the-art circuits. In addition, the radar demonstrator with scalable subarray modules simultaneously realizes range sensing and azimuth recognition for real-time pulsed radar imaging. Captured by a software-defined pulsed radar, a complete range–azimuth figure with a 1 km maximum observation range can be obtained within 150 ms under the current implementation. Users can construct a large-scale phased array radar with the presented topology of the array element and digital back-end system.

## 6. Conclusions

This paper describes the design and implementation of an X-band element-level digital phased array radar with fully integrated CMOS transceivers. Fabricated using 65 nm CMOS technology, an 8–10 GHz transceiver SoC provides analog signal processing, RF frequency translation, and global clock synchronization when the proposed periodic pulse injection technique is applied. Moreover, the scalable subarray module realizes system-level synchronization through four global reference signals and enhances the overall form factor through the use of vertically stacked printed circuit boards in a tile array configuration. Element-level digitization not only offers arbitrary weighting but also eliminates the deterministic magnitude and phase error of each radiating element. In summary, CMOS SoC integration, advanced packaging, and subarray module miniaturization result in a low-cost compact-size phased array system, and the proposed 1 × 16 subarray module accomplishes digital beamforming, back-end signal processing, and dc–dc conversion within dimensions of 317 × 149 × 74.6 ${\mathrm{mm}}^{3}$. Board-level integration of off-the-shelf front-end amplifiers further improves the performance of the 1 × 16 subarray module. Software-defined phased array radar demonstrators composed of the described subarray modules simultaneously fulfill range sensing and azimuth recognition for pulsed radar operation. A complete range–azimuth figure with a 1 km maximum distance can be captured within 150 ms by the pulsed radar demonstrator under the reported hardware implementation. In this study, waveform averaging was employed to reduce the data rate and improve the signal-to-noise ratio (SNR) for a stationary target and uncorrelated noise. The digital back-end averages out consecutively received waveforms and only delivers processed data to users. However, this technique fails to address the sensing of moving targets as a result of the time-variant relative phase in successive reflected signals. Additional hardware experiments and software algorithms should be developed for such scenarios in the future.

## Figures and Tables

**Table 1:** Performance comparison with state-of-the-art fully customized radar transceivers.

	TMTT2018 [18]	ISSCC2018 [15]	TMTT2017 [14]	TMTT2016 [16]	TMTT2016 [42]	This Work
Frequency Band	9.8–10.2 GHz	9.5–10.5 GHz	14.26–15.74 GHz	2–16 GHz	9–11 GHz	8–10 GHz
Technology	65 nm CMOS	65 nm CMOS	65 nm CMOS	0.13 μm SiGe BiCMOS	0.13 μm SiGe BiCMOS	65 nm CMOS
Die Size	1.9 × 2 mm${}^{2}$	2 × 3.9 mm${}^{2}$	1.4 × 2.9 mm${}^{2}$	2.5 × 5 mm${}^{2}$	3 × 5.2 mm${}^{2}$	2 × 2.4 mm${}^{2}$
SoC Integration	1TX + 1RX	4TX + 4RX	1TX + 1RX	8RX	1TX + 1RX	1TX + 1RX
TX Output Power	10.5 dBm	14.7 dBm	13.3 dBm	-	29.2 dBm	17.96 dBm
TX Spurious Level	-	-	-	-	-	−30.35 dBc
RX Conversion Gain	5–72 dB	Front-end: 15.3–28.6 dB Baseband: 0–60 dB	Front-end: 23.5 dB Baseband: 3–58 dB	6–11 dB	25 dB	3.8–57.2 dB
RX Noise Figure	16.5–18 dB	5.7–6.5 dB	5.6–6.3 dB	11.5–12.3 dB	3 dB	13.9–14.6 dB
RX Front-End Input P${}_{1\mathrm{dB}}$	2 dBm at 5 dB Gain −27 dBm at 32 dB Gain	−37 dBm	−33 dBm	−14 dBm	−18 dBm	−14 dBm
RX Front-End IIP${}_{3}$	7 dBm at 5 dB Gain	-	-	-	-	−5.9 dBm
RX Baseband Bandwidth	2 MHz	60–280 MHz	0.68–9.8 MHz	-	-	20 or 40 MHz
Power Consumption	147 mW	179 mW per TX 74 mW per RX	259.4 mW	250 mW per RX	4.128 W per TX 352 mW per RX	1.45 W per TX 1.41 W per RX
Number of Elements in the Demonstrator	-	4TX + 4RX	-	8RX	1TX + 1RX	16TX + 16RX
Beamforming Scheme	-	TX: RF Phase-Shifting, RX: Digital Beamforming	-	RF Phase-Shifting, Digital Beamforming	RF Phase-Shifting	Digital Beamforming
Modulation Type	Triangular Chirp	Pulsed Chirp	Sawtooth Chirp	-	-	Pulsed Chirp
Modulation Bandwidth	400 MHz (4%)	1 GHz (10%)	1.48 GHz (9.9%)	-	-	Programmable, 20 MHz for Pulsed Radar
Multibeamforming Capability	-	Yes (4 beams)	-	Yes (1, 2, or 4 beams)	-	Yes (16 beams)
Beam Steering Range	-	$\pm 60^{\circ }$	-	-	-	E-plane: $\pm 45^{\circ }$
Peak Sidelobe Ratio (PSLR)	-	−12.9 dB w/o tapering	−12.7 dB w/o tapering	-	-	−13.1 dB w/o tapering −25.6 dB w/i tapering
Radar Imaging Latency	-	off-line	-	-	-	150 ms per pulsed radar image
